# CD19嵌合抗原受体T细胞制备条件优化及其体内外杀伤作用研究

**DOI:** 10.3760/cma.j.issn.0253-2727.2022.06.011

**Published:** 2022-06

**Authors:** 春晓 任, 娴娴 陈, 莉 赵, 宇 田, 开林 徐, 恺 赵

**Affiliations:** 徐州医科大学血液病研究所、江苏省骨髓干细胞重点实验室、徐州医科大学附属医院血液科，徐州 221000 Blood Disease Institute, Xuzhou Medical University, Key Laboratory of Bone Marrow Stem Cell, Department of Hematology, the Affiliated Hospital of Xuzhou Medical University, Xuzhou 221000, China

**Keywords:** CD19, 嵌合抗原受体T细胞, B细胞淋巴瘤, CD19, Chimeric antigen receptor T cells, B-cells lymphoma

## Abstract

**目的:**

优化小鼠CD3^+^T细胞体外刺激活化体系及最佳感染时间，构建小鼠CD19嵌合抗原受体T细胞（mCD19 CAR-T），并验证其在体内外的杀伤效果。

**方法:**

磁珠分选纯化小鼠的脾脏CD3^+^T细胞，在可溶性抗CD3/CD28抗体、佛波酯+离子霉素、包被抗CD3/CD28抗体3种不同条件下刺激培养，分别于8、24、48和72 h流式细胞术检测细胞活性情况；用包含小鼠CD19抗体的scFv质粒转染Plat-E细胞，包装逆转录病毒后感染活化的CD3^+^T细胞，制备鼠特异性的CD19嵌合抗原受体T细胞（mCD19 CAR-T）。mCD19 CAR-T细胞在体外与B淋巴瘤细胞株A20细胞共培养，利用流式细胞术检测其对A20细胞的靶向杀伤效果；建立淋巴瘤小鼠模型，体内输注mCD19 CAR-T细胞，检测CAR-T细胞的体内杀伤和分布情况。

**结果:**

包被抗CD3/CD28抗体的刺激活化效果最佳，且在刺激后24～48 h具有较好的细胞活性；抗mCD19的逆转录病毒感染CD3^+^ T细胞效率［（32.27±7.56）％］稳定，制备的mCD19 CAR-T细胞可特异性杀伤A20肿瘤细胞，48 h时A20细胞凋亡率为24.3％；体内检测发现输注mCD19 CAR-T细胞第6天即可检测到脾脏中CD19^+^细胞比例［（1.83±0.58）％］显著降低，到建模后第12天骨髓和脾脏中CD19^+^细胞清除更为显著，与A20细胞组相比差异具有统计学意义［脾脏：（0.36±0.04）％对（47.00±13.46）％，*P*<0.001；骨髓：（1.82±0.29）％对（37.30±1.44）％，*P*<0.0001］。且mCD19 CAR-T细胞在外周血、脾脏和骨髓中均有分布，GFP^+^CD3^+^ CAR-T细胞比例分别为（2.90±1.12）％、（4.96±0.80）％、（13.55±1.56）％，以骨髓中占比最高。

**结论:**

获得优化的CD3^+^ T细胞活化刺激体系和最佳感染时间，稳定构建了抗mCD19 CAR-T细胞，并在体内外验证其具备良好的杀伤能力。

## Abstract (en)

**Objective:**

To optimize the stimulation and activation system of mouse CD3^+^ T cells in vitro and explore the optimal infection time of CD3^+^ T cells to establish mouse CD19 chimeric antigen receptor T cells (mCD19 CAR-T), and to also verify its killing effect in vivo and in vitro.

**Methods:**

Splenic CD3^+^T cells were isolated and purified using magnetic beads, and the cells were cultured in Soluble anti-CD3/CD28, PMA+Ionomycin, and Plated anti-CD3/CD28. Cell activation and apoptosis were assessed by flow cytometry after 8, 24, 48, and 72 hours. ScFv plasmid of mouse CD19 antibody was transfected to plat-E cells to package retrovirus. Activated CD3^+^ T cells were infected to construct mouse-specific CD19 chimeric antigen receptor T cells (mCD19 CAR-T), and mCD19 CAR-T cells were co-cultured with B-cell lymphoma cell line A20 in vitro. The specific toxicity of A20 was detected by flow cytometry, and mCD19 CAR-T cells were infused into the lymphoma mouse model to detect its killing effect and distribution.

**Results:**

The activation effect of Plated anti-CD3/CD28 on CD3^+^ T cells was superior, with the cells exhibiting good viability 24–48 hours after stimulation. Established mCD19 CAR-T cells with stable efficiency [(32.27±7.56)％] were specifically able to kill A20 tumor cells (The apoptosis rate was 24.3％ at 48 h). In vivo detection showed a non-significant decrease in the percentage [(1.83±0.58)％] of splenic CD19^+^ cells 6 days after mCD19 CAR-T cell infusion. A marked clearance in bone marrow and spleen appeared on day 12 compared with the A20 group, and this difference was statistically significant [spleen: (0.36±0.04)％ *vs* (47.00±13.46)％, *P*<0.001; bone marrow: (1.82±0.29)％ *vs* (37.30±1.44)％, *P*<0.0001]. Moreover, mCD19 CAR-T cells were distributed in high proportions in the peripheral blood, spleen, and bone marrow [(2.90±1.12)％, (4.96±0.80)％, (13.55±1.56)％].

**Conclusion:**

This study demonstrated an optimized activation system and the optimal infection time of CD3^+^ T cells. Furthermore, stable constructed mCD19 CAR-T cells showed a remarkable killing ability in vitro and in vivo.

嵌合抗原受体T细胞（CAR-T细胞）指通过载体将CAR基因转入T细胞，使其具有非主要组织相容性复合体（MHC）限制性活化、特异性杀伤肿瘤细胞功能的T 细胞。CAR结构主要由单链抗体可变区（ScFv）、铰链跨膜区和胞内信号传导结构域组成[1]。以CD19为靶点的CAR-T细胞在复发/难治的血液系统恶性肿瘤中已被广泛应用。截至2021年底，FDA先后批准多款CD19 CAR-T细胞产品Kymriah、Yescarta、Breyanzi和Tecartus分别用于治疗儿童急性淋巴细胞白血病、非霍奇金淋巴瘤（NHL）[2]–[3]和复发/难治套细胞淋巴瘤（MCL）[4]。目前，CAR-T细胞疗法被认为是最具有前景的肿瘤治疗方式之一[5]。

现有研究报道经CAR-T细胞治疗后约57％的复发/难治NHL患者达到了完全缓解，但大约60％的患者在1年内出现复发[6]。2021年Chong等[7]报道复发/难治弥漫大B细胞淋巴瘤（R/R DLBCL）经CD19 CAR-T治疗后5年的无进展生存率仅为31％。上述数据表明，虽然CD19 CAR-T细胞显著提高了复发/难治B细胞淋巴瘤患者的短期缓解率，但是长期疗效并不令人满意，肿瘤复发仍是CAR-T细胞应用中亟待解决的难题。现有研究发现CAR-T细胞在体外不能得到有效扩增是其治疗后复发的影响因素[8]，因此提高T细胞体外扩增效率以及获取最佳活化感染时间是降低CAR-T输注后复发的策略之一。

本研究运用可溶性抗CD3/CD28抗体、佛波酯+离子霉素和包被抗CD3/CD28抗体不同条件来刺激活化小鼠T细胞，获取最佳体外扩增体系和感染时间，构建mCD19 CAR-T细胞，并通过体外和体内实验验证其对CD19^+^靶细胞的杀伤效果，为后续探索CAR-T细胞输注后机体免疫应答的相关研究奠定基础。

## 材料与方法

1. 主要试剂与仪器：携带鼠CD19抗体基因和绿色荧光蛋白（GFP）的逆转录病毒质粒mCD19、携带人CD19抗体基因和GFP的逆转录病毒质粒hCD19及包装用Plat-E细胞由本实验室保存；逆转录病毒转染试剂X-tremeGENE 9 DNA transfection reagent购自瑞士Roche公司；opti-MEM和FBS均购自美国Gibco公司；DMEM高糖培养基和RPMI 1640培养基均购自江苏凯基生物技术股份有限公司；抗小鼠CD3抗体和 CD28抗体均购自美国BioGems公司；重组人IL-2购自美国PeproTech公司；小鼠T细胞分选磁珠购自美国Stem Cell公司；HitransG P病毒感染增强液购自上海吉凯基因医学科技股份有限公司；标记小鼠的CD25 PE抗体、CD3 PE抗体、CD19 BV510抗体均购自美国Biolegend公司；6～8周的雌性BABL/c小鼠购自北京维通利华实验动物技术有限公司；流式细胞仪为美国BD公司LSR Fortessa；荧光显微镜为日本Olympus公司DP72。

2. 逆转录病毒的包装：将15 µg包含mCD19或hCD19的质粒和45 µl X-tremeGENE 9 DNA transfection reagent转染试剂共同转染Plat-E细胞，转染后24、48和72 h用倒置荧光显微镜观察荧光亮度拍照；收集富含逆转录病毒颗粒的细胞上清液，0.45 µm 滤器过滤后小份分装，−80 °C保存。

3. 磁珠分选小鼠CD3^+^T细胞：获取6～8周的雌性BABL/c小鼠的脾脏，研磨获取脾脏细胞，2.5％的冰醋酸计数后，重悬至1×10^8^个/ml，根据试剂盒说明书进行磁珠分选纯化CD3^+^T 细胞。分选前后留取细胞样本，流式细胞术检测CD3^+^T细胞的纯度。

4. CD3^+^T细胞体外刺激活化：获取6～8周的雌性C57BL/6小鼠的脾脏，分选纯化后的CD3^+^T细胞用RPMI 1640培养基重悬调整细胞数2×10^6^个/ml，接种于用不同刺激剂处理的24孔板中（每孔1 ml），加入100 U/ml IL-2于37 °C、5％CO_2_孵箱培养。分组如下：①空白对照组（无刺激剂）；②可溶性抗CD3/CD28组，培养基中直接加入抗CD3和抗CD28抗体（终浓度均为2 µg/ml）；③佛波酯+离子霉素组，培养基中加入50 ng/ml 佛波酯和750 ng/ml 离子霉素；④包被抗CD3/CD28组，即利用抗CD3和抗CD28抗体包被培养板4 °C过夜（终浓度均为2 µg/ml）。不同刺激条件下刺激8、24、48和72 h 后，表面抗体CD25染色，PI区分标记死亡细胞，流式细胞术分析CD3^+^T细胞的活化及凋亡程度。

5. CAR-T细胞的制备及比例检测：CD3^+^T细胞活化36 h后，进行病毒感染。T细胞分组：①阴性对照组，即未感染病毒的活化CD3^+^T细胞；②hCD19 CAR-T组；③mCD19 CAR-T组。感染前将24孔板中的活化T细胞移入6孔板，每孔加入相应的病毒3 ml，每孔加入40 µl 1×HitransG P病毒感染增强液，1500×*g*（升降速度均为3）、30 °C离心80 min后，置于37 °C、5％ CO_2_的培养箱中继续孵育6～8 h后，更换新鲜的培养基，加入100 U/ml IL-2继续培养。荧光显微镜下观察24、48和72 h的荧光亮度。病毒感染72 h后收集细胞，流式细胞术检测GFP^+^CAR-T细胞比例。

6. A20细胞鉴定及其与CAR-T细胞共培养：淋巴瘤细胞株A20细胞表面染色CD19-BV510，流式细胞术检测A20细胞表面CD19的表达情况；将收集好的CAR-T细胞计数后与A20细胞以效靶比1∶1在24孔板中培养（每孔均5×10^5^个A20细胞），分组如下：①CD3^+^T +A20细胞组；②hCD19 CAR-T+A20细胞组；③mCD19 CAR-T+A20细胞组。流式细胞术分别检测A20细胞12、24和48 h的凋亡率。

7. 淋巴瘤小鼠模型的建立及CAR-T细胞体内杀伤作用：18只6～8周的雌性BABL/c小鼠用γ射线进行非致死剂量3 Gy照射，4 h后注射5×10^5^个A20细胞，建立淋巴瘤小鼠模型；建模后第3天注射1×10^6^个CAR-T细胞，分组如下（每组6只）：①A20细胞组；②hCD19 CAR-T+A20细胞组；③mCD19 CAR-T+A20细胞组。分别于第6和12天处死小鼠获取外周血、脾脏、骨髓，红细胞裂解后收集淋巴细胞，经CD3-PE、CD19-BV510、PI染色，流式细胞术检测CD19^+^及GFP^+^CD3^+^CAR-T细胞比例。

8. 统计学处理：流式结果使用Flowjo_V10软件进行分析，数据采用Graphpad Prism 7.0进行绘图与统计分析。两样本均数比较采用独立样本*t*检验，多个样本均数比较采用单因素方差分析，两两比较采用SNK检验。*P*<0.05为差异有统计学意义。

## 结果

1. 逆转录病毒的包装：用Plat-E细胞包装mCD19 CAR-T和hCD19 CAR-T逆转录病毒，结果可见，24 h表达绿色荧光细胞较少且荧光较弱，48 h和72 h表达绿色荧光细胞随时间增多，72 h荧光达到最强（图1）。结果表明在48 h后病毒滴度显著升高，因此收集48 h和72 h的病毒上清，以备后续实验。

**图1:**
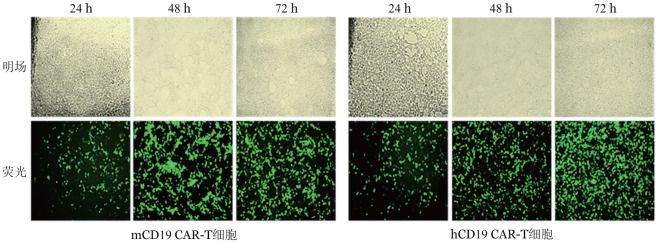
Plat-E细胞包装mCD19 CAR-T和hCD19 CAR-T逆转录病毒的明场和荧光图 CAR-T细胞：嵌合抗原受体T细胞

2. CD3^+^T细胞纯化及体外活化体系优化：经磁珠分选小鼠CD3^+^T细胞后，流式细胞术检测分选后CD3^+^T细胞的比例高达90％以上，满足后续实验要求（图2A）。CD3^+^T细胞在可溶性抗CD3/CD28抗体、佛波酯+离子霉素、包被抗CD3/CD28抗体的刺激下，与空白对照组相比，佛波酯+离子霉素组和包被抗CD3/CD28组在8 h时CD3^+^T细胞表面活化标志CD25略有升高，24 h时CD25表达显著，48 h和72 h时包被抗CD3/CD28组CD3^+^T细胞活化率可达66.4％且高于佛波酯+离子霉素组；而可溶性抗CD3/CD28组的CD3^+^T细胞随刺激时间延长仅25.8％的细胞活化，显著低于包被抗CD3/CD28组。同时检测刺激细胞的活性，结果表明佛波酯+离子霉素组和包被抗CD3/CD28组在第24 h活细胞比例开始下降，在48 h降至30％左右（图2B、2C）。上述结果说明，包被抗CD3/CD28抗体的刺激活化效果最佳，且在刺激后24～48 h具有较好的细胞活性。

**图2:**
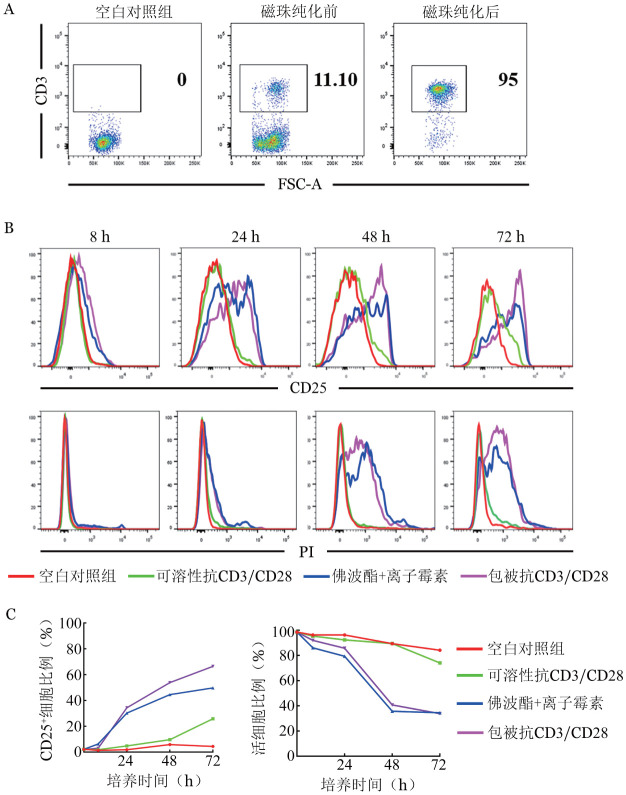
纯化CD3^+^T细胞及其最佳活化条件摸索 A：磁珠分选前后CD3^+^T细胞的纯度检测；B：CD3^+^T细胞分别在空白对照、可溶性抗CD3/CD8抗体（终浓度2 µg/ml）、佛波酯（50 ng/ml）+离子霉素（750 ng/ml）和包被抗CD3/CD28抗体（终浓度2 µg/ml）刺激下，流式细胞术检测8～72 h细胞增殖、凋亡情况；C：不同刺激条件下，8～72 h 后CD3^+^T细胞增殖、凋亡的曲线

3. CAR-T细胞的制备和培养：于活化后36 h用冻存的逆转录病毒感染，分别制备mCD19 CAR-T和hCD19 CAR-T细胞。在含有IL-2的培养基中，持续培养24、48和72 h，荧光显微镜下观察逆转录病毒感染后GFP的荧光强度。如图3A可见活化感染后T细胞变大、数量增加且聚团生长。mCD19 CAR-T细胞荧光亮度随时间逐渐增强，72 h达到最强；收集72 h感染病毒的CD3^+^T细胞，流式细胞术检测以GFP^+^为标记的CAR-T细胞比例为（32.27±7.56）％（图3B）。

**图3:**
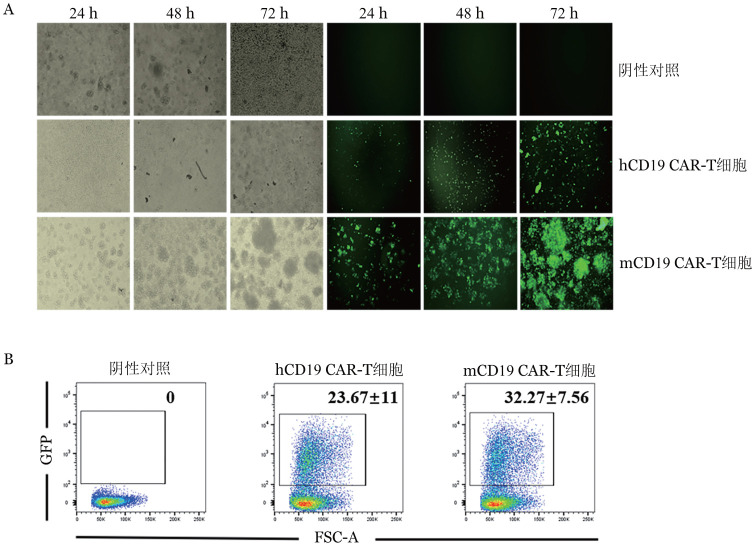
嵌合抗原受体T细胞（CAR-T）细胞制备及比例检测 A：CD3^+^T细胞感染的明场和荧光图；B：流式细胞术检测GFP^+^CAR-T细胞的比例

4. CAR-T细胞体外杀伤能力的验证：93.6％的A20细胞表达CD19分子（图4A）。CAR-T细胞与A20细胞共培养12、24和48 h后检测A20细胞的凋亡率，在第12 h mCD19 CAR-T+A20细胞组凋亡率为4.1％，与CD3^+^T+A20细胞组和hCD19 CAR-T+A20细胞组相比无明显差异；在第24、48 h，mCD19 CAR-T+A20细胞组A20细胞的凋亡率分别为8.26％和24.30％，而对照组和hCD19 CAR-T+A20细胞组无明显变化（图4B）。

**图4:**
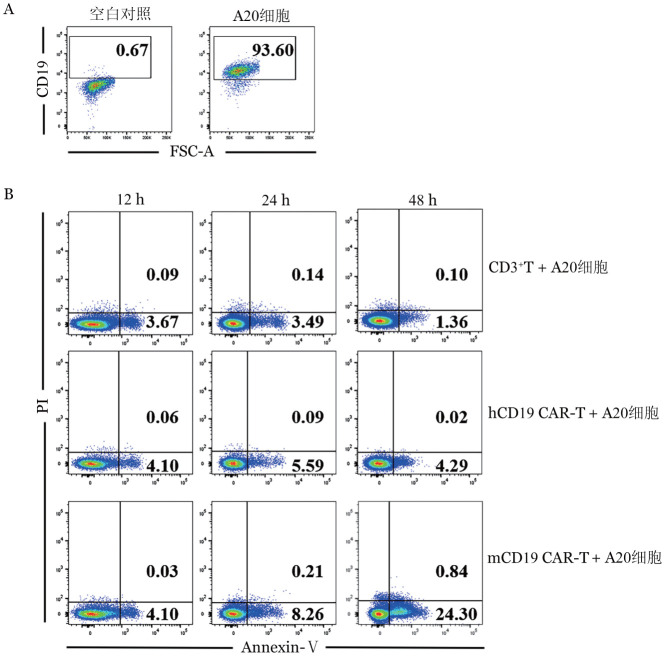
A20细胞表达CD19鉴定及CAR-T细胞对其靶向杀伤检测 A：淋巴瘤细胞株A20细胞表面CD19^+^表达率；B：未感染病毒的CD3^+^T+A20细胞组、hCD19 CAR-T组和mCD19 CAR-T组分别与A20细胞（效靶比1∶1）体外共培养12、24、48 h A20细胞凋亡率；CAR-T细胞：嵌合抗原受体T细胞

5. CAR-T细胞体内杀伤能力的验证：建模后第6天，mCD19 CAR-T组脾脏CD19^+^细胞比例［（1.83±0.58）％］显著低于A20细胞组［（12.29±2.53）％］和hCD19 CAR-T+A20细胞组［（13.22±4.83）％］，差异均有统计学意义（*P*<0.001、*P*<0.05），而外周血和骨髓中无明显差异；第12天，脾脏和骨髓中CD19^+^细胞比例均减少，显著低于A20细胞组且差异具有统计学意义［脾脏：（0.36±0.04）％对（47.00±13.46）％，*P*<0.001；骨髓：（1.82±0.29）％对（37.30±1.44）％，*P*<0.0001］，外周血中差异无统计学意义。随着时间的变化，脾脏和骨髓中A20组的CD19^+^细胞比例随时间升高，而输注mCD19 CAR-T细胞后的CD19^+^细胞比例明显下降，提示mCD19 CAR-T细胞在小鼠体内可以发挥良好的靶向杀伤作用。

6. CAR-T细胞分布的检测：建模后第12天在外周血、脾脏和骨髓中GFP^+^CD3^+^CAR-T细胞比例分别为（2.90±1.12）％、（4.96±0.80）％和（13.55±1.56）％；提示mCD19 CAR-T细胞在外周血、脾脏和骨髓中均有分布，且淋巴瘤细胞A20定植的主要器官骨髓中分布最多（图5）。

**图5:**
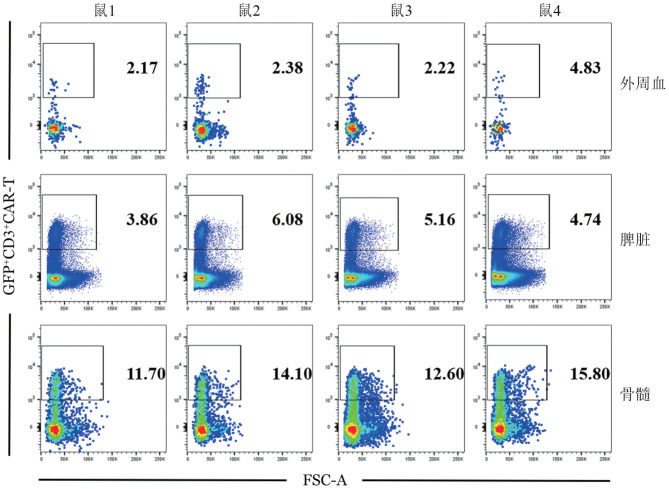
建模后第12天各组外周血、脾脏和骨髓活细胞中GFP^+^CD3^+^CAR-T细胞的分布 CAR-T细胞：嵌合抗原受体T细胞

## 讨论

CAR-T细胞疗法在肿瘤的治疗中扮演着重要的角色，在复发/难治性的血液系统恶性肿瘤中效果尤为突出。随着CAR-T细胞新的理想靶点的研发和结构优化，细胞因子释放综合征[9]、神经毒性[10]等并发症的发生率降低、程度减轻。

除了CAR的靶点和自身结构外，CAR-T细胞的疗效与T细胞的活性、扩增和感染效率有直接关系，因此本研究探索了不同刺激条件下CD3^+^T细胞活化扩增情况和最佳感染时间。对比可溶性的CD3/CD28抗体，固定化的抗体可以更有效地扩增T细胞[11]–[13]；佛波酯作为蛋白激酶C的激活物[14]和转运钙离子的离子霉素可共同激活T细胞[15]，但此活化方式显著降低了活化后T细胞的活性[16]；而且有相关研究报道T细胞在体外反复刺激活化后，亦会产生活化诱导细胞死亡（AICD），导致T细胞的活性降低，从而影响后期CAR-T细胞制备的质量[17]–[18]，所以优化T细胞刺激活化体系、探索活化细胞的最佳感染时间是实现CAR-T细胞在体内保持良好免疫状态的前提。本研究中，我们运用了可溶性抗CD3/CD28抗体、包被抗CD3/CD28抗体、佛波酯+离子霉素分别刺激活化T细胞，结果表明在包被抗CD3/CD28抗体的刺激下T细胞的克隆增殖更好，且在刺激后24～48 h仍具有良好的细胞活性。因此，采用此培养体系，可使细胞感染携逆转录病毒效率更高，且保持良好的体内免疫应答功能。

本实验利用单纯活化的CD3^+^T细胞、mCD19 CAR-T细胞和hCD19 CAR-T细胞与鼠源A20细胞共培养，活化CD3^+^T细胞和hCD19 CAR-T细胞均不能杀伤A20细胞，凋亡率无显著差异，而mCD19 CAR-T细胞对A20细胞具有显著的杀伤作用，说明了mCD19 CAR-T细胞对小鼠来源的CD19抗原具有靶向特异性。为了进一步验证mCD19 CAR-T细胞的体内效果分布特征，我们检测了A20荷瘤小鼠体内CD19^+^细胞比例。在建模后第6天和第12天，对比hCD19 CAR-T细胞组，输注了mCD19 CAR-T细胞组的小鼠在骨髓和脾脏中分别检测到CD19^+^细胞比例显著下降，表明mCD19 CAR-T细胞在体内可有效发挥特异性杀伤作用。与此同时，我们检测了CAR-T细胞在小鼠体内的分布，建模后第12天在外周血、骨髓和脾脏中，均检测到CAR-T细胞的分布，与文献[19]报道一致，且其在淋巴瘤细胞A20定植的主要器官骨髓中分布最多。

综上所述, 我们运用不同的刺激活化体系，筛选获得了最佳的CD3^+^T细胞培养条件，优化了逆转录病毒感染小鼠原代T细胞的最佳时间，成功制备了mCD19 CAR-T细胞，并在体内外展现了良好的杀伤能力，可用于我们后续进一步的研究。
